# Renal transplantation after recovery from COVID-19 - a case report with implications for transplant programs in the face of the ongoing corona-pandemic

**DOI:** 10.1186/s12882-021-02448-1

**Published:** 2021-07-06

**Authors:** Katharina Tuschen, Johanna Anders, Amin Elfanish, Verena Schildgen, Oliver Schildgen, Jan Ulrich Becker, Alexander Weidemann

**Affiliations:** 1 Medizinische Klinik III, Nephrologie und Dialyse, St. Vincenz-Krankenhaus Paderborn, Am Busdorf 2, 33098 Paderborn, Germany; 2 Medizinische Klinik I, Nephrologie, Transplantation und Internistische Intensivmedizin, Krankenhaus Merheim, Kliniken der Stadt Köln, Klinikum der Universität Witten/Herdecke, Köln, Germany; 3 Institut für Pathologie der Kliniken der Stadt Köln, Klinikum der Universität Witten/Herdecke, Köln, Germany; 4 Institut für Pathologie, Uniklinik Köln, Köln, Germany

**Keywords:** Kidney transplantation, SARS-CoV-2, Antibody response, Immunosuppression, Reactivation

## Abstract

**Background:**

The ongoing coronavirus pandemic has major impacts on both patients and healthcare systems worldwide, thus creating new realities. Patients on maintenance dialysis listed for renal transplantation are a vulnerable subgroup with many comorbidities and recurring contacts with the healthcare system. Due to the COVID-19 pandemic transplant numbers have dropped considerably, further increasing waiting times in this high-risk population. On the other hand, knowledge of the severity of SARS-CoV-2 infection in immunocompromised patients, development and persistence of neutralising antibodies in such patients is just emerging. It is unclear how best to address the dilemma of postponing the life-saving transplantation.

**Case presentation:**

We present a case report of a successful kidney transplantation only 65 days after the recipient was hospitalized for treatment of COVID-19 pneumonia. In a follow up of 9 months, we observed no signs of recurrent disease and transplant function is excellent. Monitoring SARS-CoV-2 antibody response demonstrates stable IgG levels.

**Conclusion:**

This reassuring case provides guidance to transplant centers how to proceed with kidney transplantation safely during the pandemic. Careful consideration of risks and benefits of the organ offer, full recovery from COVID-19 symptoms and the presence of a positive SARS-CoV-2 IgG antibody test, qualifies for kidney transplantation.

## Background

The rapid global spread of SARS-CoV-2 with over 115 million cases and over 2.5 million deaths of the resulting coronavirus disease (COVID-19) reported worldwide by March 2021, has led to urgent and widespread efforts to contain and mitigate transmission. The resulting disruption of many aspects of private and public life with significant socioeconomic sequelae also affected hospitals worldwide: non-life saving operations and treatment of non-life threatening illnesses were postponed. Transplant centers were also affected by various measures to contain the spread of the virus and subsequently transplant numbers fell remarkably [1].

The ongoing COVID-19 pandemic poses specific risks for waitlisted patients, patients who are already transplanted and on the transplant centers themselves: a reduced number of donated organs will affect waitlisted patients for a considerable time. Patients not yet vaccinated might defer a life-saving organ offer due to concerns over infection risks. Many hospitals, on the other hand, have their activities still not fully resumed during the second or in the face of the third wave. At this point, the number of patients who have received vaccinations varies considerably in different countries, such that many patients in need for transplantation worldwide are still forced to decide together with their physicians whether to accept an organ offer or not.

As numbers of infections are still high despite various lockdown strategies, percentages of patients vaccinated vary greatly and as mutant strains of SARS-Cov-2 spread more widely, a considerable portion of patients on dialysis will still be infected by SARS-CoV-2 with a symptomatic or sometimes asymptomatic course of COVID-19. This increasing patient cohort faces difficult decisions such as when it is safe to be transplanted or whether susceptibility for re-activation or re-infection under immunosuppressive therapy poses an unacceptable risk.

We present a patient with a successful renal transplantation 65 days after hospital discharge for COVID-19 pneumonia. Follow up of the first 100 days are very reassuring without signs of recurrent or de-novo infection. The serial detection of SARS-CoV-2 specific IgG before and after transplantation indicates that immunosuppression does not alter the B cell response to SARS-CoV-2.

## Case presentation

A 65-year-old female hemodialysis patient with ESRD due to IgA nephritis was offered a kidney from a 70-year-old deceased donor on May 27, 2020. Her waiting time was nearly 6 years. Donor eGFR was 94 ml/min, the cause of brain death was intracerebral bleeding. Additional donor risk factors were hypertension and history of smoking.

**Fig. 1:**
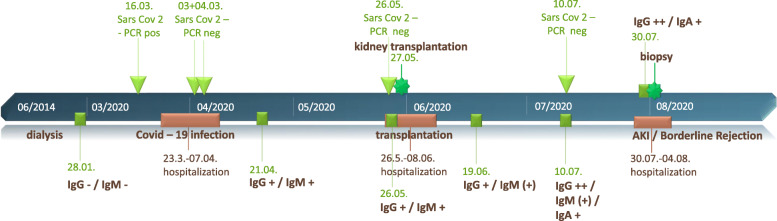
Timeline of clinical events and SARS-CoV-2 tests (PCR and serology)

The recipient herself was hospitalized for 7 days due to Covid-19 pneumonia from March 23 to April 7, 2020. Her symptoms at onset were fever and shortness of breath, and she subsequently tested positive with polymerase chain reaction (PCR) for SARS-CoV-2 in a nasopharyngeal swab on March 16, 2020 (Fig. 1). In hospital, she received nasal oxygen supplementation and supportive antibiotic treatment. High-flow O_2_-therapy or non-invasive ventilation (NIV) was not necessary. The two following PCR-tests in April were negative, and she had been discharged without respiratory symptoms 7 weeks before the organ offer. She did not report intercurrent infections.

At the time of admission for transplantation, the patient was completely symptom-free with no residual signs of infection: CRP was normal, as were leukocyte and lymphocyte counts. Chest X-ray did not show infiltrates. She again tested negative for SARS-CoV-2 by PCR, however this test result was not known until after transplantation. The transplantation into the right fossa was without surgical complications. She received our standard immunosuppressive regimen consisting of Tacrolimus, Mycophenolate and steroids after induction therapy with Basiliximab. She received Valganciclovir as her and the donor’s CMV-serostatus was positive. Her antibiotic prophylaxis was Ampicilline/Sulbactame for 7 days. The allograft developed primary function with a serum creatinine level of 1.5 mg/dl at discharge without signs of recurrent COVID-19. Laboratory results are depicted in Table 1.

**Table 1:**
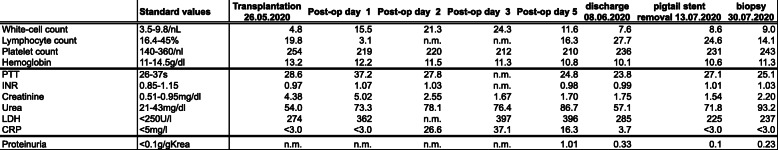
Laboratory values during transplantation and follow up (n.m. denotes *not measured)*

Eight weeks after transplantation the patient developed acute kidney injury KDIGO I. She was admitted to our hospital and again tested PCR-negative for SARS-CoV-2 in the nasal swab. The biopsy diagnosis was suspicious (Borderline) for acute T-cell-mediated rejection, mild acute tubular damage with isometric vacuolization of tubular epithelial cells, the latter suggestive of calcineurin-inhibitor toxicity at a tacrolimus trough level of 10 ng/ml. No viral particles could be found with electron microscopy in tubular epithelial cells or podocytes (not shown). Under a three-day course of high dose steroids and intravenous fluid, serum creatinine levels reached baseline again. At present, more than 9 months after transplantation, the patient has an uncomplicated follow-up with a stable serum creatinine of 1.6 mg/dl.

### SARS-CoV-2 PCR testing

At the onset of COVID-19 pneumonia, the patient tested PCR-positive for SARS-CoV-2 on March 16, 2020 (Fig. 1). Subsequently, the nasopharyngeal swab became negative on April 2nd. Between this sample and the sample before transplantation, no tests were performed as the patient did not have typical symptoms of COVID-19. At time of transplantation in May, PCR was still negative, as were the following tests which were done before scheduled admissions to the hospital (Fig. 1).

### Analysis of antibody response

**Fig. 2:**
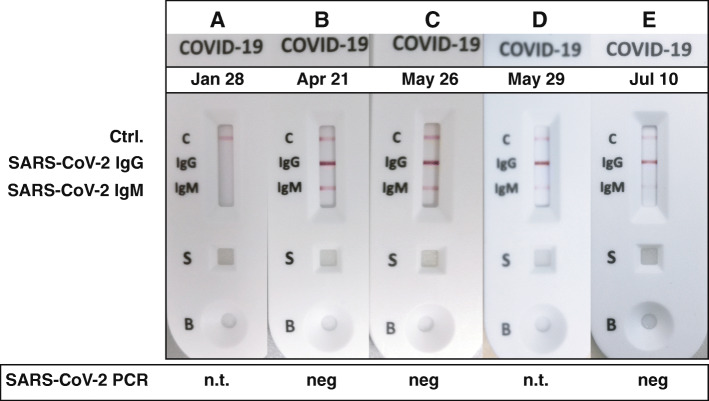
Serial tests of the IgG and IgM response after infection with SARS-CoV-2. Sample (**A**) was from routine HLA-antibody testing on the waitlist before infection, (**B**) after recovery from COVID-19 pneumonia, demonstrating a typical antibody response. **C** was at admission for transplantation with persistent IgG levels, which were merely unchanged even after full induction therapy (**D**). **E** depicts the gradual loss of IgM with stable IgG response 6 weeks after kidney transplantation. Samples (**A**) to (**C**) were re-analyzed after the patient had been admitted, thus results were not known at the time of transplantation. Samples (**D**) and (**E**) were analyzed prospectively. PCR tests were negative at times indicated (n.t. denotes *not tested*)

For a complete overview of the antibody reaction to SARS-CoV-2, we retrospectively analyzed plasma which was assessed on the waiting list before she suffered COVID-19 pneumonia. While SARS-CoV-2 PCR in the throat wash and nasal swabs remained negative on all tests since convalescence, antibody testing for IgG and IgM showed a classic serological response (Fig. 2). As can be seen, SARS-CoV-2 antibodies were not detectable in January, where the routine HLA-testing was performed before her infection with SARS-CoV-2. After returning on the waiting-list the serum of April already shows an antibody response. Directly before and after transplantation in May, IgG detection remains stable with decreasing intensity of IgM detection, a trend continuing until July.

### Renal biopsy

Renal function declined on July 30 during follow up, due to biopsy-proven Borderline rejection and possible mild CNI-toxicity. Despite lack of symptoms of upper respiratory tract infection, we wanted to rule out COVID-19 associated renal injury. Electron microscopy did not show viral particles (not shown).

## Discussion and conclusions

This is one of the few cases of kidney transplantation after COVID-19. In contrast to the first report [2], our case has distinct clinical features, which gives important guidance for future handling of the persistent pandemic situation worldwide: first, our patient had a more severe course of COVID-19 pneumonia which required hospitalization. Even after such a relatively severe course of COVID-19, transplantation and immunosuppression appear to be safe. As numbers of COVID-19 patients rise, more and more dialysis patients will have survived COVID-19 and will be eligible for transplantation. Second, serial detection of SARS-CoV-2 IgG and IgM clearly demonstrates an intact and ongoing B cell response despite immunosuppressive therapy. Moreover, presence of IgG antibodies after infection in dialysis patients is also reassuring, since immune responses are known to be diminished in this patient population. In contrast to our data, a recent case report of a simultaneous pancreas-kidney transplant patient showed loss of an IgG response with persistent total antibodies (IgG and IgM) [3]. The meaning of this observation in contrast to our and other reports is unclear to this date.

To this date, it is not known whether SARS-CoV-2 can actually recur. There have been several reports [4–6], however as serological responses were not reported potential recurrences could be actually persistent infections in which PCR was falsely negative [7–9]. Some reports documented re-infection with a phylogenetically distinct virus [10], indicating that precautionary measures such as masks and distancing still have to be taken. In two newer case reports transplantation was performed without complications in patients with a positive PCR exhibiting an asymptomatic or mild course [11, 12]. Although the favorable outcomes are reassuring, more data is clearly needed to stratify individual risks and benefits of organ transplantation in a certain clinical setting. In our case, under immunosuppressive therapy, the absence of clinical symptoms and the repeatedly negative tests ruled out persistent or recurrent infection. Unsurprisingly, there was no involvement of the graft with SARS-CoV-2 infection, given the repeatedly negative PCR tests.

A strength of our study is the longitudinal documentation of the serologic response: IgG and IgM-antibodies against SARS-CoV-2 were still detectable weeks after transplantation. Gradual weakening of IgM is normal for an antibody response. As the test is not quantitative and it is not known whether the antibodies display neutralizing properties, this finding has to be interpreted with caution. Interestingly, SARS-CoV-2 infection on primates has been shown to induce immunity [13], which is corroborated by the clinical parameters of our patient. Although we did not monitor T cell responses, the IgG antibody persistence and the clinical follow-up would argue for the relative safety of anti-rejection therapy after COVID-19.

Our study has several limitations: obviously, the follow-up time is relatively short. A substantially longer time will be necessary to draw conclusions regarding patient and graft survival and whether COVID-19 associated complications might occur. However, with the level of immunosuppression highest at the time of transplantation, infectious complications are expected to emerge at this point in time; also because the recovery from COVID-19 was only recent. From a clinical perspective, as already described in other case reports, we did not know the antibody status at time of reperfusion. This has prompted us to implement screening strategies on the waiting list, which facilitates decision making at the time of the organ offer.

This case has several important implications for transplant centers worldwide.

First, it demonstrates that transplantation and immunosuppressive therapy is possible already 7 weeks after SARS-CoV-2 infection. We conclude that this is reassuring for patients who have suffered from a more severe course of COVID-19 even with hospitalization. As Massie et al. have demonstrated, there are only very few scenarios when postponing transplantation is - from the patients’ perspective - warranted [14] especially in the light of organ shortage, long waiting times and excessive mortality on the waiting list.

Taken together, our case suggests that, after careful consideration of risks and benefits of the organ offer, full recovery from COVID-19 symptoms and the presence of a positive SARS-CoV-2 IgG antibody test qualifies for kidney transplantation. Even though knowledge of the efficacy of the antibody response is currently not clearly defined in the dialysis population, we suggest patients on the waiting list for kidney transplantation should undergo routine serologic testing for SARS-CoV-2. In consideration of the ongoing pandemic and the protracted vaccination programs globally, this might facilitate decision making at the center level to proceed with transplantation.

## Data Availability

All data generated or analysed for this case presentation are included in this published article [and its supplementary information files].

## Abbreviations

CMV: Cytomegalovirus
CNI: Calcineurin inhibitor
COVID-19: Coronavirus disease 2019
CRP: C reactive protein
eGFR: Estimated glomerular filtration rate
ESRD: End stage renal disease
HLA: Human leukocyte antigen
Ig A/M/G: Immunoglobulin A/M/G
KDIGO: Kidney Disease: Improving Global Outcomes
NIV: Non-invasive ventilation
PCR: Polymerase chain reaction
SARS-CoV-2: Severe acute respiratory syndrome coronavirus type 2
